# Determination of the Enhancement or Shielding Interaction between Two Parallel Cracks under Fatigue Loading

**DOI:** 10.3390/ma12081331

**Published:** 2019-04-24

**Authors:** Zhichao Han, Caifu Qian, Lanqing Tang, Huifang Li

**Affiliations:** Department of Chemical Mechanics Engineering, Beijing University of Chemical Technology, Beijing 100029, China; hanzhichaohzc@163.com (Z.H.); lihf@mail.buct.edu.cn (H.L.)

**Keywords:** parallel cracks, crack interaction, finite element analysis, fatigue

## Abstract

In this paper, the interactions between two parallel cracks are investigated experimentally and numerically. Finite element models have been established to obtain the stress intensity factors and stress distributions of the parallel cracks with different positions and sizes. Fatigue crack growth tests of 304 stainless steel specimens with the single crack and two parallel cracks have been conducted to confirm the numerical results. The numerical analysis results indicate that the interactions between the two parallel cracks have an enhancement or shielding effect on the stress intensity factors, depending on the relative positions of the cracks. The criterion diagram to determine the enhancement or shielding effect between two parallel cracks is obtained. The changes of the stress fields around the cracks have been studied to explain the mechanism of crack interactions.

## 1. Introduction

Fatigue damage of ships, aircrafts, pressure vessels, and other engineering components will be caused by fluctuation loadings during their service time [1]. The accumulation of the fatigue damage of engineering components leads to fatigue cracks [2]. Generally, multiple cracks can be found in the damaged components [3]. Compared with the single crack, multiple cracks experience the interactions and thus affect the remaining strength of damaged components [4]. Therefore, it is of great importance to investigate the interactions between multiple cracks.

Previous studies have investigated multiple crack interactions and their effects on the stress intensity factor [5,6,7,8,9,10,11,12,13,14]. Kamaya [5,6] performed the linear–elastic and elastic–plastic analysis by the finite element method for the interactions between the semicircular and semi–elliptical surface cracks under a tensile or bending load, and obtained the relationship between the magnitude of the interactions and the relative positions of the cracks. Ma et al. [7] studied the interactions between an edge and an embedded parallel crack, and they found that the normal and deviation distances as well as the relative crack sizes could affect the value of the stress intensity factors of the two cracks. Kishida et al. [8] investigated the priority of propagation among three parallel cracks, and they found that the longest crack did not always have the maximum value of the stress intensity factor due to the crack interactions. Jiang et al. [9] studied two unequal parallel cracks in a finite width plate subjected to a remote tensile load. They found that because of the crack interactions, the stress intensity factors at the tips of two cracks simultaneously decreased. It was also found that when the difference between the lengths of the two cracks was high, the short crack was dormant, and its influence could be neglected. Moussa et al. [10,11,12] studied the interactions between two non–coplanar, semi–elliptical surface cracks and calculated the stress intensity factors of the cracks as a function of the crack front position, depth, shape, and plate thickness. An empirical formula was derived, which was related the effect of the relative positions of these cracks to the stress intensity factors. Meng et al. [13] investigated the crack interactions between two parallel cracks and obtained the influence coefficients of the different relative distance between the cracks. Isida et al. [14] researched the relations between stress intensity factors and the crack number, and proposed reliable formulations of the stress intensity factors for collinear and parallel cracks under various load conditions.

It is widely acknowledged that the stress intensity factor is an important parameter to characterize the intensity of the stress field at the crack tip. Over the years, numerous methods and models have been proposed to calculate the stress intensity factors of the cracks of different configurations [15,16,17,18,19,20,21,22,23,24]. Cartwright et al. [15] presented a versatile method to obtain stress intensity factors of complex configurations. They divided the complex configurations into some simple configurations, and the stress intensity factors of the complex configurations could be compounded by those of the simple configurations. Kuang et al. [16] proposed an iterative method based upon the principle of superposition and the ideas of self–consistency to obtain the stress intensity factors of two parallel cracks. However, this method was not valid when the distance between the two cracks was too close. Kachanov et al. [17] proposed an approximate method to obtain the stress intensity factors of multiple cracks based on the principle of superposition. This method decomposed the traction of each crack into a uniform component and a non-uniform component, and the non-uniform component could be neglected. Therefore, this method was also not valid when the distance between the two cracks was too close. Based on the Kachanov method, Li et al. [18,19] decomposed the traction of each crack into a linear component and a nonlinear component, and Qing et al. [20] considered the non–uniform component of the traction of cracks through the alternating technique. These two methods were valid particularly for the situation when two cracks were close. Based on the superposition principle of the elasticity theory, Xiao et al. [21] obtained an analytical elastic solution for the stress intensity factors of the cracks when the distance between the two cracks was larger than the crack length. Moreover, they calculated the stress intensity factors of two penny–shaped cracks with different sizes in a three–dimensional elastic solid under the uniaxial tension situation. Based on the superposition principle and integral equations, Graham et al. [22,23,24] calculated the stress intensity factors of two penny–shaped cracks in an infinite or finite solid under the normal and shear loads.

Recently, several researchers have investigated the effects of the crack interactions on the fatigue crack behavior [25,26,27,28,29]. It was found that the crack interactions could influence the crack growth path and crack growth rate of materials. Jiang et al. [25] studied the fatigue propagation behavior of two parallel edge–cracks in a finite plate. They found that the cracks had a tendency to go away from the original propagation path and this tendency increased with the increasing crack length and decreasing crack distance. Hui et al. [26] found that the crack growth rates of multiple cracks deviated from those of the single crack. They introduced a new parameter Δ*K*_n_ as a new driving force for fatigue growth based on the net section stress range Δ*σ*_n_. Kamaya [27,28,29] studied the interactions between two parallel surface cracks by means of the fatigue tests and finite element method. They found that the inner crack tips of the two surface cracks changed their growth direction so that they approached each other. The growth direction of the outer crack tips was almost straight, perpendicular to the load direction.

It is commonly accepted that two adjacent cracks may interact with each other in terms of the enhancement or shielding effect. As the geometric configuration with two cracks is complicated, no accurate theoretical solutions for their interactions are available except collinear cracks. In this study, the interactions between two parallel cracks were investigated both numerically and experimentally. Concentration was placed on the determination whether and how the stress intensity factors of the two cracks were affected.

## 2. Numerical Simulations

### 2.1. Geometrical Model

**Figure 1:**
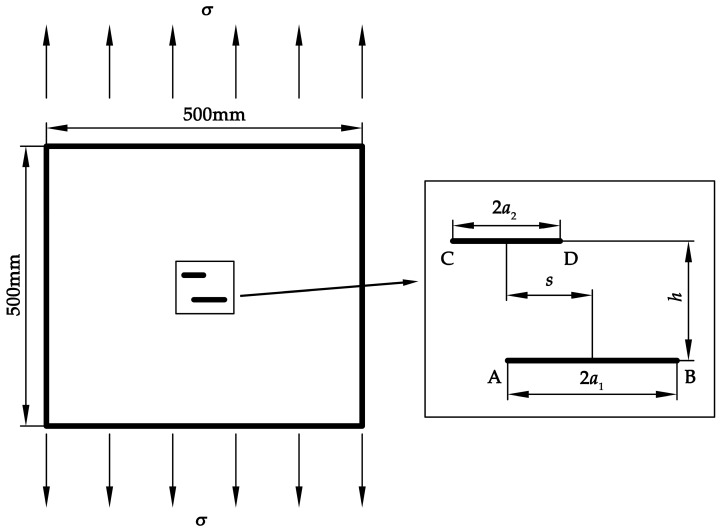
Geometric model of a plate with two parallel cracks.

A finite element model of a plate with two through-thickness parallel cracks under uniform remote tension *σ* of 125 MPa is established, as shown in Figure 1. The plate model is 500 mm × 500 mm × 6 mm in size. The length of the long crack and the short crack is 2*a*_1_ and 2*a*_2_, respectively. The ratio of the short crack length to the long crack length, *a*_2_/*a*_1_, is denoted by *R_a_*, and the long crack length *a*_1_ is 3 mm. Since the crack size is significantly smaller than the plate size, the plate can be considered as an infinite plate. Particularly, as shown in Figure 1, the crack tips of the long crack and the short crack are represented with symbols A, B, C, and D. The deviation and normal distances between the two cracks are denoted by *s* and *h*, respectively. Specially, if the deviation distance *s* equals zero, two cracks share the same perpendicular bisector. If the normal distance *h* equals zero, two cracks are considered to be collinear. The material adopted in this model is 304 stainless steel with the Young’s modulus of 195 GPa and the Poisson’s ratio of 0.3 [30]. Linear–elastic analysis is performed to calculate the stress intensity factors at the crack tips.

### 2.2. Mesh Model

**Figure 2:**
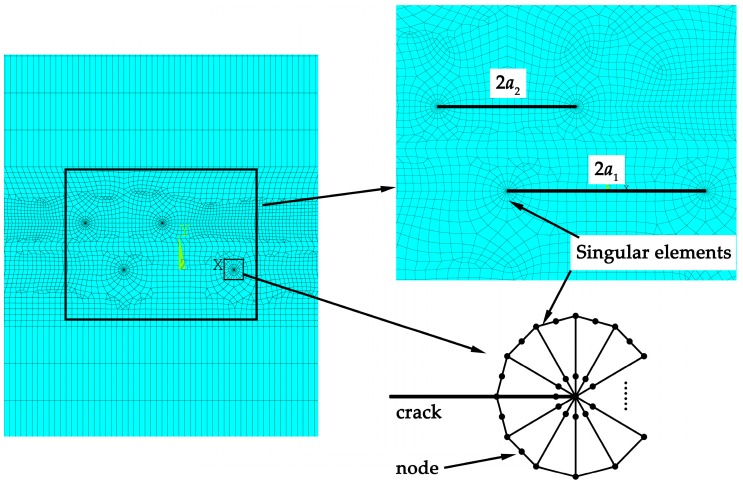
Mesh model of the plate with two parallel cracks.

The eight-node plane element, with the software ANSYS (version 18.0, ANSYS Inc, Pennsylvania, U.S.A) is used to generate meshes. In the region around the crack tips, meshes are refined to improve the calculation accuracy as shown in Figure 2. The stress intensity factors of the crack tips are calculated by the displacement extrapolation method [31]. A special command in ANSYS, the KSCON (key point stress concentration) command, is executed to generate the singular elements [32] at the crack tips. The midnodes near the crack tip of the singular elements are skewed to the 1/4 point. The dimension of singular elements at crack tips is 1/20 of the crack length. The PLANE 183 element is chosen and twenty singular elements are created at each crack tip. The total numbers of elements and nodes are 85,799 and 258,460, respectively. 

### 2.3. Simulation Results of the Interactions between the Parallel Cracks

#### 2.3.1. Stress Intensity Factors at the Crack Tips

**Figure 3:**
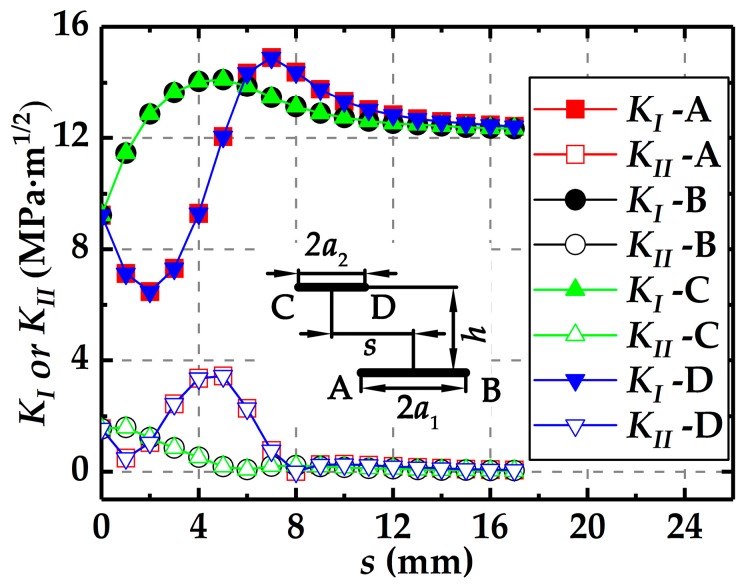
Changes of *K*_I_ or *K*_II_ with *s* at *h* = 2.5 mm for *R_a_* = 1.0.

As shown in Figure 1, the plate model is subjected to the uniform tensile loading. Figure 3 illustrates the changes of stress intensity factors at the four crack tips with the increasing *s* at *h* = 2.5 mm for *R_a_* = 1.0. Although both the two cracks are mixed mode I and II, it can be seen from Figure 3 that the mode II stress intensity factor, *K*_II_, is much smaller than the mode I stress intensity factor, *K*_I_, for a given *s* and *h*. Therefore, only *K*_I_ is considered to evaluate the interactions between the parallel cracks. In addition to the crack sizes, the crack relative distances, i.e., the deviation *s* and normal distance *h*, should be considered to affect the crack interactions.

**Figure 4:**
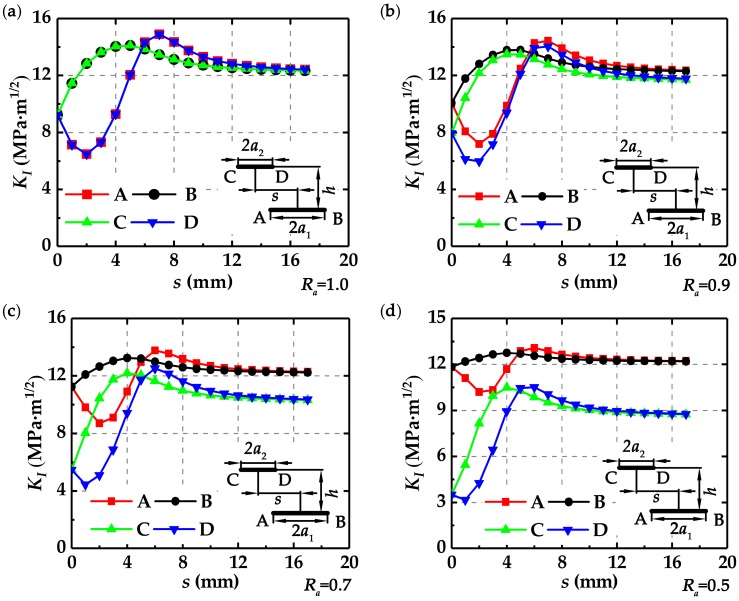
Changes of *K*_I_ with *s* at *h* = 2.5 mm for different *R_a_*: (**a**) *R_a_* = 1.0, (**b**) *R_a_* = 0.9, (**c**) *R_a_* = 0.7, and (**d**) *R_a_* = 0.5.

Figure 4 shows that the stress intensity factors at the four crack tips change with crack length ratio *R_a_* and deviation distance *s* at *h* = 2.5 mm.

It is found that for different *R_a_*, the stress intensity factor *K*_I_ at tips A and D shows the same trend, as shown in Figure 4. Initially, *K*_I_ at tips A and D decreases with the increasing *s* and falls to the minimum values. Then *K*_I_ increases sharply and reaches the maximum values. After that, *K*_I_ gradually decreases, and finally becomes stable. The stress intensity factor *K*_I_ at tips B and C also shows the same trend. Initially, *K*_I_ at tips B and C increases with the increasing *s* and reaches the maximum values. Then, *K*_I_ gradually decreases and finally becomes stable. From Figure 4a to Figure 4d, it is observed that *K*_I_ at tips C and D decreases with the decreasing *R_a_* at the same *s*. In addition, for the same *s* but different *R_a_*, the value of *K*_I_ at the crack tip C is always less than that at the crack tip B, and the value of *K*_I_ at the crack tip D is always less than that at the crack tip A, implying that the long crack is more “dangerous” than the short crack.

**Figure 5:**
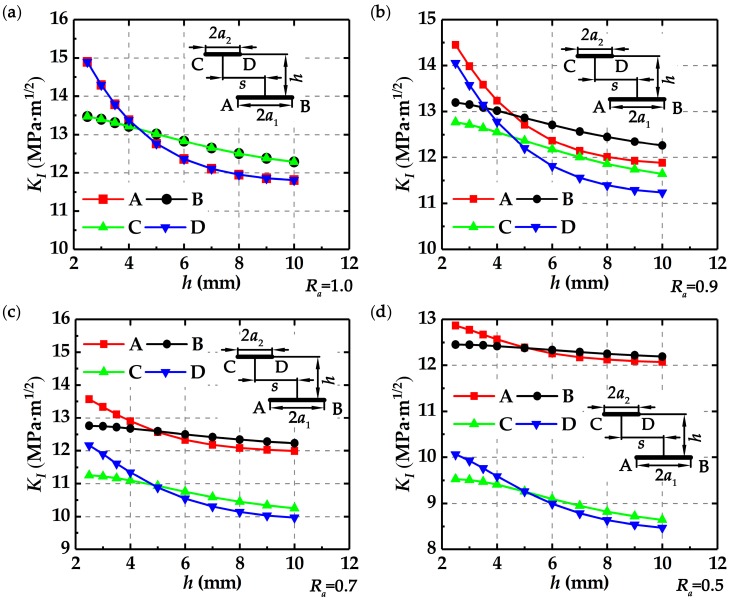
Changes of *K*_I_ with *h* at *s* = 7 mm for different *R_a_*: (**a**) *R_a_* = 1.0, (**b**) *R_a_* = 0.9, (**c**) *R_a_* = 0.7, and (**d**) *R_a_* = 0.5.

Figure 5a–d show the changes of *K*_I_ with the increasing *h* at *s* = 7 mm for *R_a_* = 1.0, *R_a_* = 0.9, *R_a_* = 0.7, and *R_a_* = 0.5, respectively. It seems that *K*_I_ at tips A and D decreases almost linearly with the increasing *h*, while *K*_I_ at tips B and C decreases in a parabola manner with the increasing *h*, implying that *h* affects the near crack tips and remote crack tips to different degrees. In addition, *K*_I_ decreases with the decreasing *R_a_* for the same *h*, which means that the relative crack sizes influence their interactions. It is also found that the value of *K*_I_ at the short crack tip is less than that at the long crack tip, and the difference of *K*_I_ between two cracks increases with the decreasing *R_a_*. 

In order to illustrate the enhancement or shielding effect of the two parallel cracks more clearly, a single crack is modeled as the reference. The stress intensity factor at the single crack tip is denoted by *K*_I_^0^. The ratio of stress intensity factor of the parallel cracks to the stress intensity factor of the single crack, *K*_I_/*K*_I_^0^, is introduced to characterize the crack interactions. Specially, if the value of *K*_I_/*K*_I_^0^ is more than one, the crack interaction is considered to be enhanced, and if the value of *K*_I_/*K*_I_^0^ is less than one, the crack interaction is shielded.

As indicated before, for the two parallel cracks with different lengths under the fatigue loading, the long crack is usually regarded as to be more "dangerous". Thus, in the following analysis, we focus on the effect of the short crack on the stress intensity factors of the long crack.

**Figure 6:**
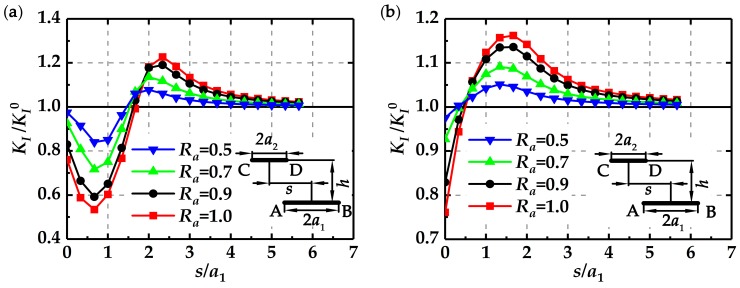
Changes of *K*_I_/*K*_I_^0^ with *s*/*a*_1_ at *h* = 2.5 mm for different *R_a_*: (**a**) *K*_I_/*K*_I_^0^ at tip A, and (**b**) *K*_I_/*K*_I_^0^ at tip B.

Figure 6 shows the values of *K*_I_/*K*_I_^0^ at tips A and B changing with *s*/*a*_1_ at *h* = 2.5 mm for different *R_a_*. It is observed that if the value of *s*/*a*_1_ is small, the corresponding value of *K*_I_/*K*_I_^0^ is less than one, which means that the influence of the short crack on the long crack is shielding. As *s*/*a*_1_ increases, the value of *K*_I_/*K*_I_^0^ increases to be larger than one, meaning that the shielding effect of short crack turns into the enhancement effect. When the value of *s*/*a*_1_ is more than five, the value of *K*_I_/*K*_I_^0^ approaches one, indicating that the interactions between two cracks vanish. These results illustrate that the deviation distance between two parallel cracks plays a key role in the crack interactions. As shown in Figure 6, it is observed that the crack length ratio, *R_a_*, also affects the crack interactions. Actually, a larger *R_a_* tends to pose a greater enhancement or shielding effect.

#### 2.3.2. Determination of the Enhancement, Shielding, or no Interaction Effect between Cracks

It is of great importance in engineering and academic research if we can determine the enhancement, shielding, or no interaction effect between the cracks without concrete numerical calculation. To achieve this goal, a large number of numerical simulations with different crack configurations are carried out. Here, for the convenient judgment of numerical computation, it is set that if the value of *K*_I_/*K*_I_^0^ is greater than 1.025, the stress intensity factor of the crack is considered to be enhanced and if *K*_I_/*K*_I_^0^ is smaller than 0.975, the stress intensity factor of the crack is considered to be shielded. Otherwise, the crack interactions are neglected or in other words, the cracks do not interact with each other.

**Figure 7:**
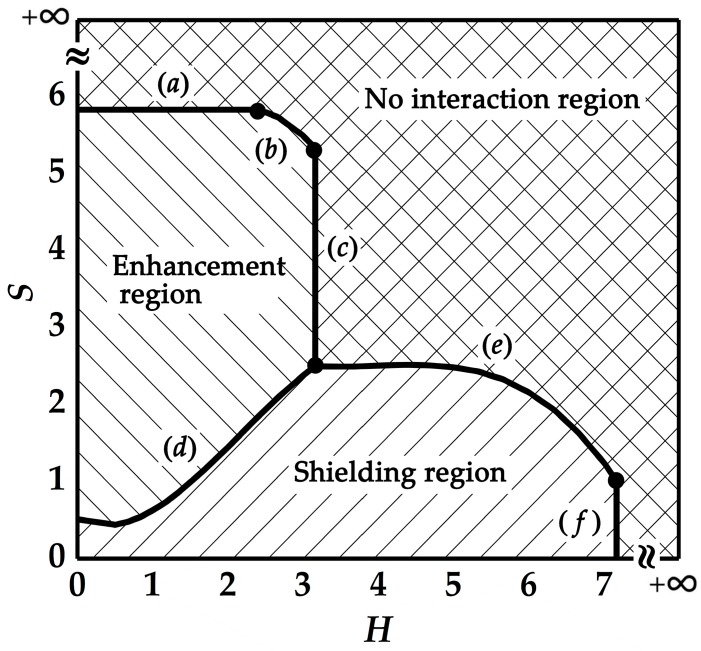
Criterion diagram for the interactions between two parallel cracks.

With sufficient numerical results, the criterion diagram to determine the enhancement, shielding, or no interaction effect between two parallel cracks is obtained, as shown in Figure 7. To make the criterion expression more concise and universal, two dimensionless numbers *H* and *S* are introduced. Here, *H* represents the ratio of the normal distance to the half of the crack length, i.e., *h*/*a*, and *S* represents the ratio of the deviation distance to the half of the crack length, i.e., *s*/*a*. Specially, to determine the effect of the short crack on the long crack, *a* in *H* and *S* is the half of the short crack length, *a*_2_. Likewise, to determine the effect of the long crack on the short crack, *a* in *H* and *S* is the half of the long crack length, *a*_1_.

The expressions of the boundaries of a–f can be obtained by the least square method [33]. The diagram is divided into three regions by these boundaries.

The enhancement region can be express by the inequalities shown in Equations (1) and (2):

$$\left\{ \begin{array}{lll} -0.100H^{3}+0.655H^{2}-0.440H+0.511\leq S<5.833 & & \\ & \;\mathrm{for}\;0\leq H<2.333 & (1)\\ -0.100H^{3}+0.655H^{2}-0.440H+0.511\leq S<-0.600H^{2}+2.703H+2.795 & & \\ & \;\mathrm{for}\;2.333\leq H<3.167 & (2) \end{array}\right.$$

The shielding region can be express by the inequalities shown in Equations (3) and (4):

$$\{\begin{array}{cc} 0\leq S<-0.100H^{3}+0.655H^{2}-0.440H+0.511\;\mathrm{for}\;0\leq H<3.167 & (3)\\ 0\leq S<-0.049H^{3}+0.579H^{2}-2.256H+5.385\;\mathrm{for}\;3.167\leq H<7.167 & (4) \end{array}$$

Accordingly, the no interaction region can be express by the inequalities shown in Equations (5)–(8):

$$\{\begin{array}{llll} S\geq 5.833 & \;\mathrm{for}\; & 0\leq H<2.333 & (5)\\ S\geq -0.600H^{2}+2.703H+2.795 & \;\mathrm{for}\; & 2.333\leq H<3.167 & (6)\\ S\geq -0.049H^{3}+0.579H^{2}-2.256H+5.385 & \;\mathrm{for}\; & 3.167\leq H<7.167 & (7)\\ S\geq 0 & \;\mathrm{for}\; & H\geq 7.167 & (8) \end{array}$$

From Figure 7 it can be found that if the two cracks are close and share the same perpendicular bisector, i.e., *s* = 0, only the shielding effect exists. This result implies that for two cracks sharing the same perpendicular bisector, it would be too conservative and even irrational to simply merge them into a bigger crack by applying the enveloping method, or in other words, it is safe to just consider the long crack.

On the other hand, if the two cracks are close and collinear, i.e., *h* = 0, only the enhancement effect exists. Of course, when the two cracks are not close, either in deviation or in normal distance, their interactions can be neglected. 

It is noted that in Figure 7, both *S* and *H* are dimensionless, and this means that the determination of the enhancement or shielding effect of the two parallel cracks is independent of the absolute length of the cracks. This result is of importance in engineering since it can be applied in the practical structures with the similar multi–crack configurations.

## 3. Experiments

### 3.1. Specimen Preparation

The hot–rolled plates of 304 stainless steel are machined into the suitable dimensions (260 mm × 48 mm × 6 mm). The chemical composition (wt%) of the steel is listed in Table 1 [34].

**Figure 8:**
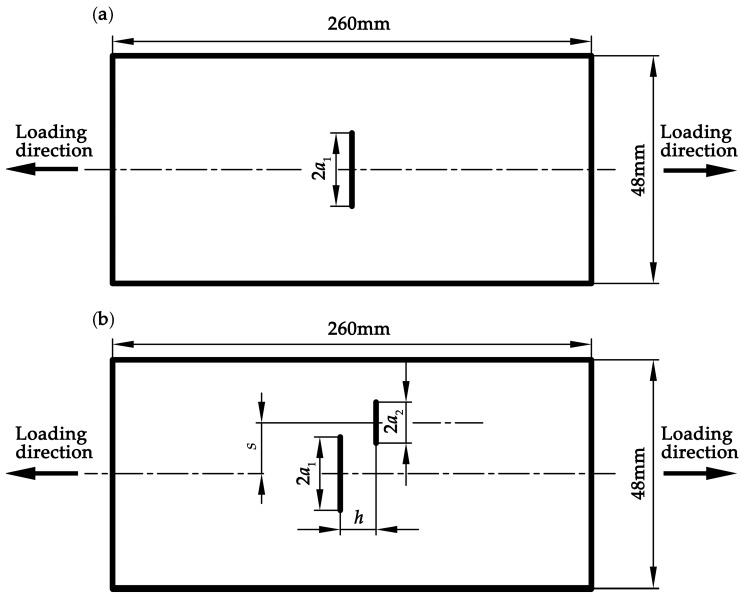
Geometry of the test specimens: (**a**) the single crack specimen; and (**b**) the parallel crack specimen.

The through-thickness notches are made using the wire electrical discharge method, and the diameter of the wire used is 0.2 mm. Table 2 lists the positions and sizes of the notch cracks in different specimens. In order to verify the crack interactions studied in the above section, five specimens are specially designed, namely the single crack specimen (SC), the parallel crack specimen with *R_a_* = 0.9 and *s* = 0 (PC0.9S0), the parallel crack specimen with *R_a_* = 0.9 and *s* = 7 (PC0.9S7), the parallel crack specimen with *R_a_* = 1.0 and *s* = 0 (PC1.0S0), and the parallel crack specimen with *R_a_* = 1.0 and *s* = 7 (PC1.0S7), as shown in Figure 8.

### 3.2. Settings of the Fatigue Test

**Figure 9:**
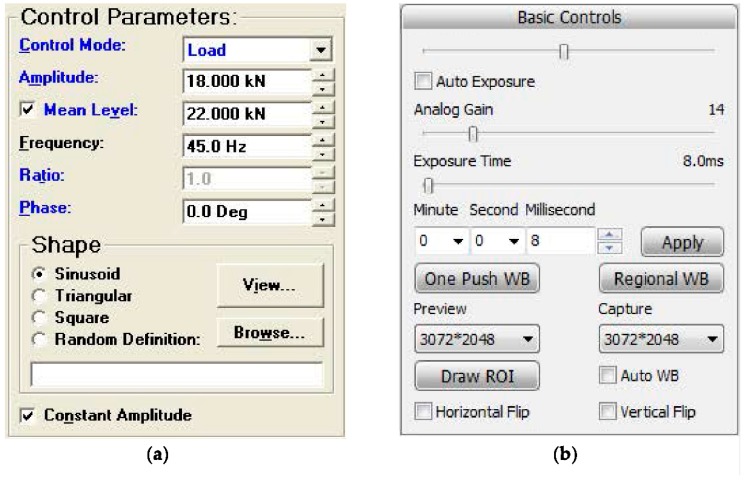
Experimental setups: (**a**) the INSTRON 8800 fatigue testing machine; and (**b**) the digital microscope system.

An INSTRON 8800 fatigue testing machine with the Single Axis MAX software (Boston, Massachusetts, U.S.A) is used to carry out the fatigue crack growth tests. A constant amplitude load with stress ratio *R* of 0.1, the maximum load of 40 kN, and loading frequency of 45 Hz is employed. A digital microscope system is used to monitor and record the crack length during the fatigue tests. The experimental setups are shown in Figure 9.

### 3.3. Results of the Tests

#### 3.3.1. Crack Growth Paths

**Figure 10:**
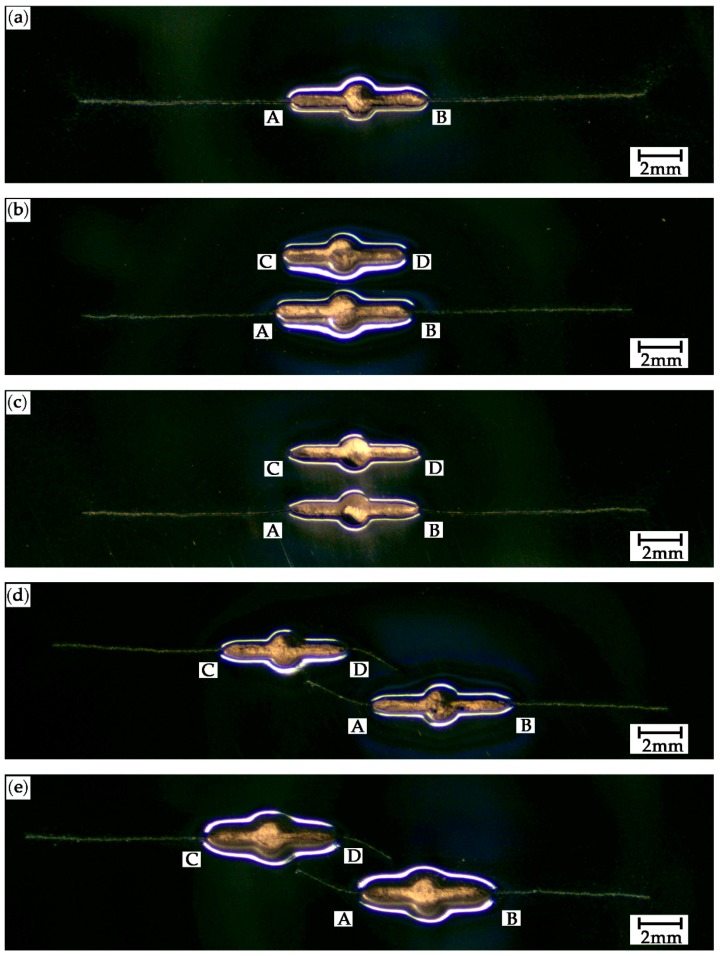
Crack growth paths in different specimens: (**a**) SC, (**b**) PC0.9S0, (**c**) PC1.0S0, (**d**) PC0.9S7, and (**e**) PC1.0S7. Points A-D are the four crack tips shown in Figure 1.

Figure 10a–e show the crack growth paths in the SC, PC0.9S0, PC1.0S0, PC0.9S7, and PC1.0S7 specimens, respectively.

For the SC specimen, as shown in Figure 10a, the paths are perpendicular to the loading direction. For the PC0.9S0 and PC1.0S0 specimens, the crack paths of the tips A and B are perpendicular to the loading direction but the cracks do not grow at the tips C and D due to the shielding effect caused by the adjacent crack, as shown in Figure 10b,c. For the PC0.9S7 and PC1.0S7 specimens, the crack growth paths of the tips B and C are perpendicular to the loading direction, but the cracks growth paths of the tips A and D are no longer perpendicular to the direction of the loading, clearly also because of crack interactions, as shown in Figure 10d,e.

#### 3.3.2. Stress Intensity Factors

The stress intensity factors at the crack tips along the crack growth paths are calculated numerically. Corresponding to the range of the fatigue load, both the Mode I stress intensity factor range, Δ*K*_I_, and the Mode II stress intensity factor range, Δ*K*_II_, are obtained.

**Figure 11:**
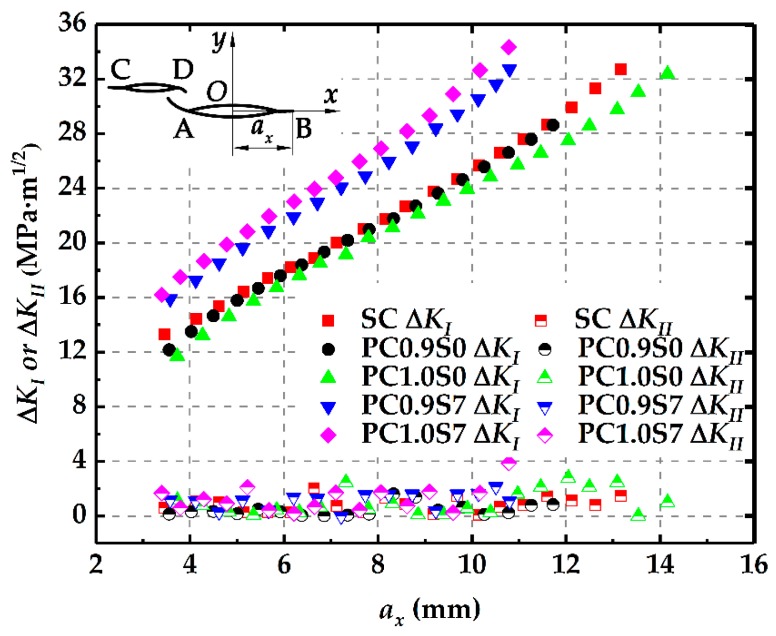
Changes of Δ*K*_I_ and Δ*K*_II_ at tip B with the increasing *a_x._*

Figure 11 shows Δ*K*_I_ and Δ*K*_II_ at the crack tip B changing with the horizontal growth length *a_x_* in different specimens. Clearly, Δ*K*_I_ increases almost linearly with the increasing *a_x_* for all the specimens. Δ*K*_II_, however, fluctuates around a very small value, which means that the cracks propagate in Mode I. Compared with Δ*K*_I_ in the single crack, Δ*K*_I_ at tip B in the two parallel cracks with the deviation distance (PC0.9S7 and PC1.0S7) increases significantly while that in the two parallel cracks without the deviation distance (PC0.9S0 and PC1.0S0) decreases in some extent. Obviously, these results are consistent with those obtained in Section 2.3.1.

#### 3.3.3. Crack Growth Rates

**Figure 12:**
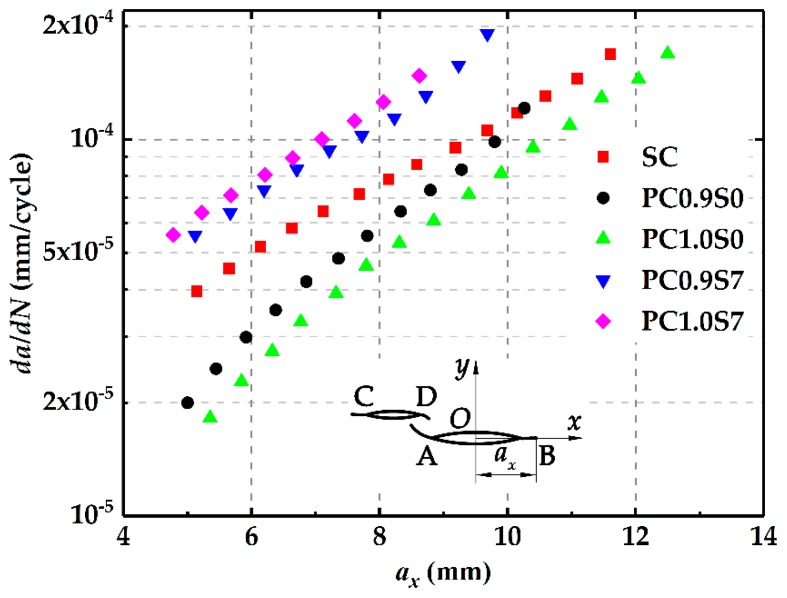
Changes of crack growth rates at tip B with the increasing *a_x._*

Figure 12 shows crack growth rates at the crack tip B changing with the horizontal growth length *a_x_* in different specimens. It is found that at the same *a_x_*, the crack growth rates in the PC0.9S7 and PC1.0S7 specimens are higher than those in the SC specimen. In contrasts, the growth rates in PC0.9S0 and PC1.0S0 specimens are lower than those in the SC specimen. For the specimens with the same deviation distance, the crack growth rates in the PC1.0S7 specimen are larger than those in the PC0.9S7 specimen, while the crack growth rates in the PC1.0S0 specimen are smaller than those in the PC0.9S0 specimen. Combined with the simulation results in Section 2.3, it can be found that crack growth rates are influenced by the enhancement or shielding effect. Specifically, the crack growth rates in the parallel crack specimen increase with the increasing enhancement effect while decrease with the increasing shielding effect.

## 4. Discussion on the Mechanism of the Crack Interactions

It seems that the two parallel close cracks present their interactions in two opposite ways. One is that the crack causes material discontinuity, thereby weakening the stress field around cracks. The other is effective crack length, which is defined as the overall projected length of the cracks on the surface perpendicular to the first principal stress. The increase of the effective crack length can strengthen the stress field around the cracks. How the two cracks interact with each other depends on the resultant effect of the two influences. Of course, if the two cracks are remote from each other, i.e., a large *s* or *h*, the stress field is not considered to be affected. 

**Figure 13:**
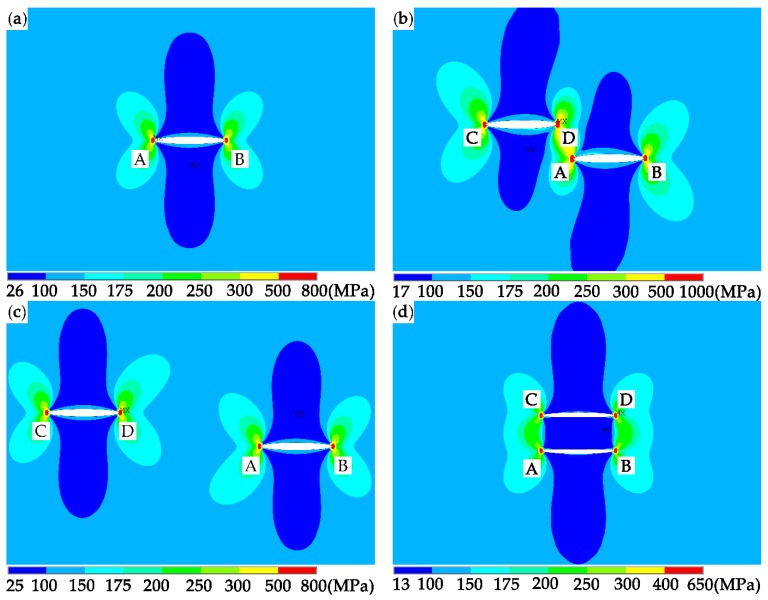
Von Mises stress distributions in the vicinity of the crack tips: (**a**) SC, (**b**) PCS7, (**c**) PCS17, and (**d**) PCS0. Points A-D are the four crack tips shown in Figure 1.

**Figure 14:**
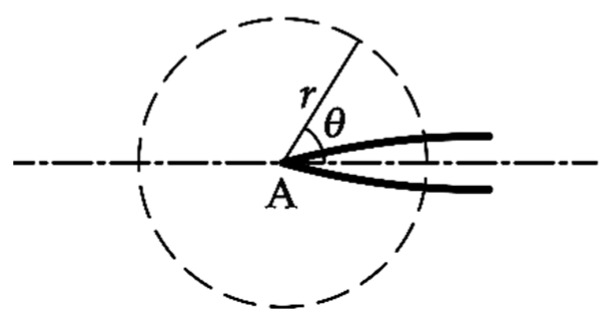
A circle defined to show stress distributions.

To prove this viewpoint, the changes of the stress fields around the cracks caused by crack interactions are obtained. Figure 13 shows the stress distributions in the vicinity of the crack tips for the single crack (SC), the two equal parallel cracks with *s* = 7 and *h* = 2.5 (PCS7), *s* = 17 and *h* = 2.5 (PCS17), and *s* = 0 and *h* = 2.5 (PCS0). In order to avoid the stress singularity at the crack tip, a circle with the center at tip A and the radius, *r*, of *a*_1_/10 is chosen, as shown in Figure 14, to compare the stress distributions in the vicinity of tip A for different crack configurations.

**Figure 15:**
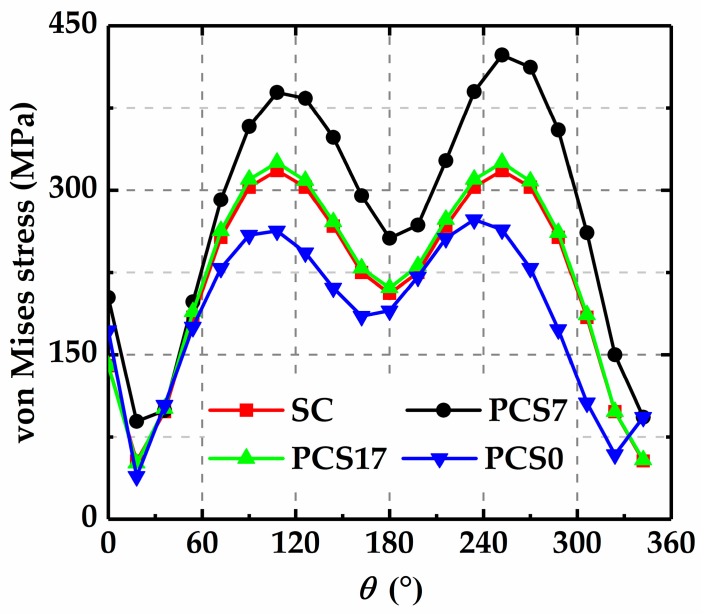
Von Mises stress distributions in the vicinity of tip A.

Figure 15 shows the stress distributions in the vicinity of the crack tip A for the SC, PCS7, PCS17 and PCS0. It is found that the stress magnitude in the vicinity of tip A of PCS7 is larger than that of SC, while the stress magnitude of PCS0 is smaller, compared with that of SC. For PCS17, however, since the two cracks are far deviated from each other, the stress field is not clearly affected. These results indicate that if the two parallel cracks are close and deviated, the stress field can be strengthened, and if the two parallel cracks are close and share the same perpendicular bisector, the stress field is weakened.

## 5. Conclusions

In this paper, the interactions in terms of enhancement or shielding between two parallel cracks with different sizes and positions have been investigated numerically and experimentally. Conclusions are obtained as follows:

1. If the two parallel cracks are close and share the same perpendicular bisector, only the shielding effect exists. In this case, it would be too conservative and even irrational to simply merge them into a bigger crack by applying the enveloping method.
2. If the two parallel cracks are close and deviated, whether the stress intensity factors are enhanced or not depends on the deviation and normal distance between the two cracks. Specifically, if the two parallel cracks are collinear, only the enhancement effect exists.
3. The criterion diagram to determine the enhancement, shielding, or no interaction effect between two parallel cracks is obtained, which can be applied in practical structures with similar multi-crack configurations.
4. Fatigue crack growth test results indicate that the cracks grow in Mode I. The crack growth rates are influenced by the enhancement or shielding effect. Specifically, the crack growth rates in the parallel crack specimens increase with the increasing enhancement effect while decrease with the increasing shielding effect.
5. The crack interaction phenomenon can be explained by the changes of the stress fields around cracks. If the two parallel cracks are close and deviated, the stress field is strengthened and if the two parallel cracks are close and share the same perpendicular bisector, the stress field is weakened.

## Figures and Tables

**Table 1:** Chemical composition of S30408 (wt.%).

Material	C	Mn	P	S	Si	Cr	Ni
304	≤0.08	≤2.00	≤0.045	≤0.03	≤1.00	18.0–20.0	8.0–10.5

**Table 2:** Positions and sizes of the notch cracks in the specimens.

		The Shielding Effect		The Enhancement Effect	
	SC	PC0.9S0	PC1.0S0	PC0.9S7	PC1.0S7
*a*_1_ (mm)	3	3	3	3	3
*a*_2_ (mm)	—	2.7	3	2.7	3
*s* (mm)	—	0	0	7	7
*h* (mm)	—	2.5	2.5	2.5	2.5
